# Biomarkers for Alzheimer's Disease in Saliva: A Systematic Review

**DOI:** 10.1155/2019/4761054

**Published:** 2019-05-02

**Authors:** Helena Sophia Gleerup, Steen Gregers Hasselbalch, Anja Hviid Simonsen

**Affiliations:** Danish Dementia Research Centre, Department of Neurology, Rigshospitalet Copenhagen University Hospital, Denmark

## Abstract

**Background:**

The histopathological changes of Alzheimer's disease (AD) are detectable decades prior to its clinical expression. However, there is a need for an early, inexpensive, noninvasive diagnostic biomarker to detect specific Alzheimer pathology. Recently developed neuroimaging biomarkers show promising results, but these methods are expensive and cause radiation. Furthermore, the analysis of cerebrospinal fluid (CSF) biomarkers requires an invasive lumbar puncture. Saliva is an easily obtained body fluid, and a stable saliva biomarker would therefore be a promising candidate for a future method for diagnosing AD. The purpose of this systematic review was to investigate studies of biomarkers in saliva samples for the diagnosis of AD.

**Methods:**

The included articles were identified through a literature search in PubMed and Google Scholar for all articles until November 1^st^, 2018, and furthermore, all reference lists of included articles were reviewed by hand. We included articles written in English investigating saliva from patients with AD and a control group.

**Results:**

A total of 65 studies were identified, whereof 16 studies met the inclusion criteria and were included in the systematic review. A plethora of different biomarkers were investigated, and ten out of the sixteen studies showed a statistical significance in biomarkers between patients with AD and healthy, elderly controls, among these biomarkers for specific AD pathology (amyloid beta 1-42 (A*β*42) and tau).

**Conclusion:**

A*β*42 and tau seem to be worthy candidates for future salivary biomarkers for AD, but other biomarkers such as lactoferrin and selected metabolites also have potential. More studies must be carried out with larger sample sizes and a standardization of the sampling and processing method. Factors such as diurnal variation, AD patients' decreased ability of oral self-care, and salivary flowrates must be taken into consideration.

## 1. Introduction

Alzheimer's disease (AD) is a neurodegenerative disease and is the leading cause of progressive dementia. It is estimated that 46.8 million people suffer from dementia worldwide, with the highest prevalences found in the older age groups (+65 years). By 2030, it is estimated that the prevalence will increase to approximately 74.7 million people, partly due to the increasing numbers of elderly people in the world [1]. AD is the cause of approximately 60% - 80% of dementia-related cases in people over 65, while it is only 30-40% in people under 65 years [2]. Cognitive deficits in AD progress with the duration of the disease caused by accelerating neurodegenerative processes. Formation of specific AD pathology, amyloid plaques between neurons and the accumulation of intracellular neurofibrillary tangles composed of tau, begin decades prior to the clinical expression of AD, and it is therefore essential to find a biomarker for early preclinical diagnosis and treatment monitoring. The biomarker sampling and analysis must be easy to perform, inexpensive, and noninvasive. Currently, an analysis of the cerebrospinal fluid (CSF) is used to aid the diagnosis of AD, which later in the disease course has good diagnostic precision [3]. Changes in the CSF's content of tau, phosphorylated tau, and amyloid beta 1-42 (A*β*42) can at this point be detected in most patients [4]. There is currently no disease-modifying treatment available for AD, but numerous trials are ongoing, especially in presymptomatic or early symptomatic stages of the disease [5]. Very recently, the monoclonal antibody, Aducanumab, was abandoned in phase III due to an analysis which concluded that Aducanumab would not be able to slow the cognitive decline by decreasing the production and aggregation of A*β*. Furthermore, it was reported that antiamyloid agents might not have a clinical effect in the symptomatic stages of the disease [6, 7]. Therefore, it is essential to develop a sensitive and noninvasive method for early diagnosis and monitoring, so a potentially disease-modifying intervention can be initiated in the presymptomatic or prodromal phase and thereby delay the onset of AD or modify the disease course.

Saliva is an easily obtained body fluid, and studies have reported that proteins from the central nervous system (CNS) are excreted into the saliva [8]. Many parts of the body are affected by AD, among these parts of the autonomic nervous system (ANS), including the brain stem, the hypothalamus, the cerebral neocortex, the insular cortex, and locus coeruleus. In addition, studies have reported that AD degenerates nerve terminals in the cholinergic system, which regulates the cardiovascular system and the ANS and that this alteration already can be seen in the preclinical phase of the disease [9]. The submandibular, the sublingual, and the parotid glands, which are the main salivary glands in the mouth, secrete saliva in response to cholinergic innervation from the glossopharyngeal cranial nerve and the facial cranial nerve, controlled by ANS. Consequently, an alteration in the ANS, as seen in AD, could affect the saliva production and composition, and this altered composition might thereby mirror pathological changes in the CNS [10]. In addition, studies have shown that most blood biomarkers can also be found in saliva, and it has been reported that proteins from the blood can pass into the saliva via passive diffusion, active transport, or microfiltration [8, 10, 11]. Furthermore, it has been suggested that some AD biomarkers, such as A*β*42, are expressed or produced in the salivary glands [10, 12, 13]. As a result, a saliva sample could be a valid alternative to CSF or blood, because the saliva sampling is easy to perform, inexpensive, and noninvasive. A valid and reproducible saliva biomarker would therefore be preferable over other present biomarkers. For this reason, the purpose of this systematic review was to review the studies of biomarkers obtained in saliva for AD diagnosis.

## 2. Methods

### 2.1. Eligibility Criteria

Studies selected for review included original, full-text articles published in English, investigating biomarkers for AD in saliva. Studies must include saliva samples from AD patients and a control group.

### 2.2. Search

The original studies were identified through a literature search in PubMed and Google Scholar for all relevant articles up until November 1^st^, 2018. The filters “English” and “humans” were applied, and the following keywords were used for the search: (Saliva) AND diagnos^∗^ AND (Alzheimer OR AD) AND (biomarker). Furthermore, all reference lists of identified studies were reviewed by hand.

### 2.3. Study Selection and Data Extraction

By screening the titles and the abstracts based on the eligibility criteria listed above, studies were selected for further data extraction. The selected original, full-text articles were reviewed independently by the first and last author according to a developed data extraction sheet, including biomarker identification, age and gender for AD patients and controls, inclusion and exclusion criteria, diagnostic criteria, analytical method, blinding of analysts, and statistics for the biomarkers involved in the study.

## 3. Results

**Figure 1:**
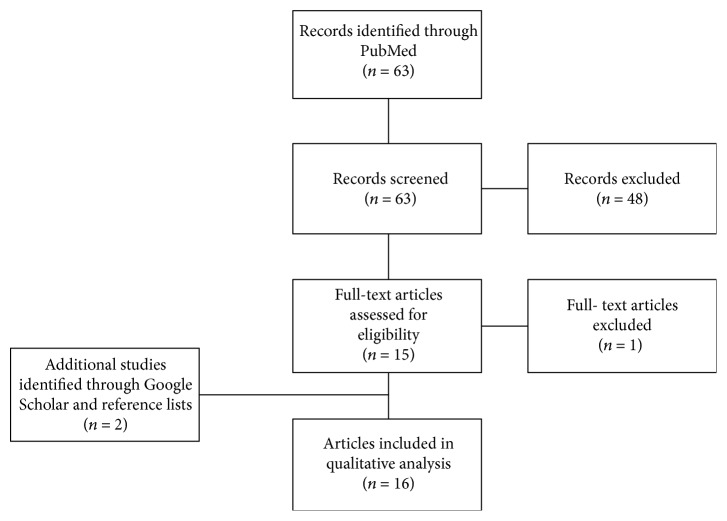
Flowchart of the literature search and the study selection.

The study selection process is seen in Figure 1. The initial literature search in PubMed identified 63 studies. The titles and the abstracts from all identified studies were screened, and 48 studies were excluded, either due to irrelevance or because the studies did not fulfil the inclusion criteria. The remaining fifteen original studies were evaluated in full, and fourteen studies met the inclusion criteria and were therefore included in the qualitative analysis. In addition, a literature search in Google Scholar and a review of the reference lists of all included studies identified two further studies, which were included in the qualitative analysis. Altogether, a total of sixteen original, full-text articles were included in the systematic review.

### 3.1. Preanalytical Variables

Sampling and processing methods varied among studies. Seven studies required fasting prior to saliva sample collection [14–20], and eight studies required rinsing of the mouth before the saliva sample collection [14–18, 21–23]. In nearly all of the studies, the saliva collection was performed as unstimulated by spitting or drooling directly into a tube, except for one study where the sample collection was executed with a Salivette [24]. In three studies, the method for the saliva collection was not described [12, 13, 18]. The volume of saliva sample collected varied from 1 mL to around 5 mL.

### 3.2. Saliva Biomarkers

Biomarkers from the sixteen included articles were divided into the following categories: *β*-amyloid, tau, acetylcholinesterase, and other biomarkers.  Table 1 shows an overview of the studies. Statistically significant difference in biomarker concentrations between the patients with AD and the control group was found in ten out of sixteen studies and will be described below.

#### 3.2.1. *β*-Amyloid

A*β*42 and A*β*40 in saliva were investigated in seven studies, which altogether included 187 subjects with AD, 72 subjects with Parkinson disease (PD), and 195 healthy controls. In four studies, increased A*β*42 levels in patients with AD were detected with enzyme-linked immunosorbent assay (ELISA) [12, 13, 20, 25]. In addition, one of these studies found an interaction with age (*p* value = 0.016) and an interaction with gender (*p* value = 0.002) [20]. An additional study used an immunoassay with nanobeads to detect an increased A*β*42 level with statistical significance, but no *p* value was provided [21]. Two other studies also used ELISA but did not detect A*β*42 in the saliva samples [19, 24]. Two studies reported no statistical significance on A*β*40 concentrations [20, 21].

#### 3.2.2. Tau

Phosphorylated tau (p-tau) and total tau (t-tau) in saliva were investigated in four studies, which altogether included 181 subjects with AD, 123 subjects with amnestic mild cognitive impairment (aMCI), twenty subjects with PD, sixteen subjects with frontotemporal dementia (FTD), and 317 healthy controls. An increased p-tau/t-tau ratio in AD patients was identified with ELISA (*p* value < 0.05) in one study [24]. Furthermore, one of the studies reported an increased p-tau/t-tau ratio using a Western blot analyzing phosphorylation sites S396, S404, T404, and a combination of S400 and T403 (*p* value < 0.05) and an increased median p-tau/t-tau ratio at phosphorylation site S396 (*p* value < 0.05) [26]. In the two remaining studies, ELISA [19] and single molecule array (SIMOA) [15] were used to detect p-tau and t-tau. Although both p-tau and t-tau levels were described as increased in the two studies, no statistical significance was reported.

#### 3.2.3. Acetylcholinesterase (AchE) Activity

AchE activity in saliva was investigated in three studies, which altogether included 66 subjects with AD, thirteen subjects with vascular dementia (VaD), and 39 healthy controls. All three studies were performed by Ellman's colorimetric method. In one study, decreased AchE activity was identified in patients with AD (*p* value < 0.005), and an interaction with age in healthy controls was reported (*p* value < 0.001) [23]. In contrast, increased AchE activity was reported in one study but with no statistical significance [16]. No statistically significant difference between patients with AD and healthy controls was found in the last study [22].

#### 3.2.4. Other Biomarkers

Other biomarkers in saliva (lactoferrin, selected metabolites, and trehalose) were investigated in five studies, which altogether included 430 subjects with AD, 102 subjects with aMCI, 79 subjects with PD, and 426 healthy controls. In one study, decreased levels of lactoferrin were detected with ELISA both in AD patients compared to healthy controls (*p* value < 0.001) and in aMCI patients compared to healthy controls (*p* value < 0.001). In addition, the study identified a positive correlation with CSF A*β*42 and t-tau (*p* value < 0.001) and a positive correlation with minimental state examination (MMSE) in AD patients compared to aMCI patients (*p* < 0.001) [27].

Within the group of metabolites, one study detected increased levels of propionate among AD patients (*p* value < 0.034) [17] by using the proton NMR spectroscopy, while another study reported increased levels of spinganine-1-phosphate, ornithine, and phenyllactic acid (*p* value < 0.01) and decreased levels of inosine, 3-dehydrocarnithine, and hypoxanthine (*p* value < 0.01) by using the fast ultraperformance liquid chromatography mass spectrometry (FUPLC-MS) [18]. The third study used liquid chromatography mass spectrometry (LC-MS) for investigating metabolites in saliva. The study found a statistically significant difference (*p* value < 0.01) between AD patients and the healthy control group in methylguanosine, histidylphenylalanine, choline-cytidine, phenylalanylproline, phenylalanylphenylalanine, and urocanic acid. The study also found a statistically significant difference (*p* value < 0.01) between AD patients and aMCI patients in the metabolites: amino-dihydroxybenzene, glucosyl-galactosyl-hydroxylysine-H_2_O, aminobytyric acid + H_2_, alanylphenylalanine, and phenylalanylproline [14].

Finally, increased levels of trehalose in AD patients as compared to controls were found in one study by using an extended gate ion-sensitive field-effect transistor biosensor (EG-IDFET biosensor) but with no statistical significance [19].

## 4. Discussion

Biomarkers obtained from the CSF is a well-established method used to detect AD pathology, but the procedure is invasive and complications and adverse effects are frequently encountered [28]. Simultaneously, early diagnosis is essential for the prognosis, disease monitoring, and treatment of AD [29]. As a result, it is crucial to find a method that is easy and safe to perform, inexpensive, and noninvasive and is able to detect biomarkers in the presymptomatic phase. The purpose of this systematic review was to assess the existing literature on salivary biomarkers for AD. Although the exact source that excretes biomarkers from the CNS into the saliva is still undefined, saliva has shown to be a valid candidate for detection of biomarkers in AD. Studies have reported that most of the compounds found in blood can also be found in saliva, and consequently, it has been suggested that compounds from the blood can pass into the saliva via passive diffusion, active transport, or microfiltration [8, 10, 11]. Furthermore, it has been proposed that biomarkers are excreted directly from the axons of the glossopharyngeal cranial nerve and the facial cranial nerve that stimulate the salivary glands. In addition, it may be possible that biomarkers are expressed or produced in the salivary glands [10]. Studies have shown that, for example, A*β*42 is produced by all organs, which can serve as the explanation to why A*β*42 is increased in saliva when it is decreased in the CSF of patients with AD [12, 13].

The results obtained on salivary A*β*42 indicate that A*β*42 is a good candidate for a future salivary biomarker. Five out of seven studies reported increased levels of salivary A*β*42 in AD patients [12, 13, 20, 21, 25], while two studies did not detect A*β*42 [19, 24]. Common to all studies were a small sample size, and for that reason, further studies must be carried out with more participants. Two studies reported on statistical insignificant difference in concentrations of A*β*40 [20, 21], resulting in a less reliable salivary biomarker when compared to A*β*42. Further studies should also investigate p-tau and t-tau as salivary biomarkers. Only two out of four studies found a statistically significant increase [24, 26] in p-tau and t-tau, although the remainder of the studies also reported nonsignificantly increased levels in AD [15, 19]. The lack of concordance between the four studies might be due to the fact that four different techniques were used to analyze the saliva samples or because of a lack of sensitivity of the assays used. For that reason, a standardization of sampling and analytical methods must be performed in order to confirm the validity of p-tau and t-tau as salivary biomarkers. The results found on AchE activity in AD patients indicate that AchE activity is not a promising salivary biomarker for early detection of the disease [16, 22, 23], although AchE activity could serve as an indicator for the pathology of AD or as an indicator of the degeneration of the cholinergic system. Bakhtiari et al. [16] suggests that further studies must investigate the AchE activity according to the severity of the patients' disease (mild, moderate, and severe) and furthermore consider a standardization of the study design. In addition, it should be considered that different variables, such as sex, AchE inhibitor therapy, delirium, and stress, can affect AchE activity [16, 30, 31].

Besides these main biomarkers for AD, other biomarkers in saliva were investigated. The results found by Carro et al. [27] indicate that lactoferrin is a promising candidate for a future salivary biomarker for AD. Lactoferrin is an iron-binding glycoprotein, and it is one of the most important antimicrobial peptides in saliva. Lactoferrin increases the activity of leukocytes, it is bacteriocidic, and by functioning as an antioxidant, it can protect the body against free radicals (ROS). Furthermore, lactoferrin is antiviral by inhibiting viral receptors, which results in an inhibited binding between virus and healthy cells. Studies have shown that bacteria and viruses are involved in the pathology of AD by altering the permeability of the blood-brain barrier and thereby facilitating an overproduction and aggregation of A*β*42 [27, 32, 33]. For that reason, more studies must be performed in order to verify if decreased levels of lactoferrin can serve as an early, salivary biomarker for AD. In one study, trehalose was examined. Trehalose is a sugar molecule, and it is believed to be associated with physiological and metabolic changes in the body and therefore the pathophysiology of AD [19]. In addition to trehalose, four exploratory studies examined a plethora of different metabolites using four different unbiased analytical methods for AD biomarker discovery. Many of the metabolites were either significantly increased or decreased in AD patients [14, 17, 18]. Therefore, more studies must be conducted in order to verify which metabolites reflect the pathology of AD best and subsequently investigate if the results are reproducible.

Salivary biomarkers for the diagnosis of AD are still a new research area in need of more studies. A*β*42, tau, lactoferrin, and different metabolites seem to be worthy candidates for future salivary biomarkers for AD. However, the source that excretes the biomarkers into the saliva is still undefined. Based on the results of this systematic review, many of the included studies used different detection techniques. It is therefore essential to standardize the sampling, processing, and analytical methods with the purpose of investigating the reproducibility of the results. In addition, larger sample sizes must be considered, and thereby the reference intervals of the biomarker's concentration can be assessed. Another aspect, which should be taken into consideration, is diurnal variation of the biomarkers. Six out of the sixteen included full-text articles took the possible diurnal variation into account [15, 16, 18, 20, 22, 26], which is essential to avoid that a normal circadian rhythm affects the results. Furthermore, AD patients' decreased ability of self-care raises the question if a decreased oral health or hygiene could affect the detection of the biomarkers. It should also be taken into account that studies have shown AD patients have a decreased saliva production either due to side effects of the medication (antidepressants and antipsychotics) or due to the pathology of the disease [34]. Therefore, future studies must evaluate biomarker levels taking salivary flowrates and the oral health or hygiene into account to ascertain the clinical usefulness of saliva for early diagnosis of AD.

## Figures and Tables

**Table 1:** An overview of the subjects and on the results of the included articles.

References	Biomarkers investigated	Methods used to analyze biomarkers	Number of subjects	Age of subjects	Sex of subjects	Result biomarkers
Sabbagh et al. [25]	A*β*42	ELISA	AD: *n* = 15 Healthy controls: *n* = 7	Mean age AD: 77.8 ± 1.8 Healthy controls: 60.4 ± 4.7	Male/female AD: 7/8 Healthy controls: 2/5	↑ A*β*42, *p* value AD/control < 0.05

Huan et al. [14]	Metabolites	LC-MS	Two studies (discovery and validation) *Discovery* AD: *n* = 22 aMCI: *n* = 25 Healthy controls: *n* = 35 *Validation* AD: *n* = 7 aMCI: *n* = 10 Healthy controls: *n* = 10	Two studies (discovery and validation) *Discovery* Mean age AD: 77.09 aMCI: 70.4 Healthy controls: 69.94 *Validation* Mean age AD: 70.11 aMCI: 71.5 Healthy controls: 71.4	Two studies (discovery and validation) *Discovery* Male/female AD: 6/16 aMCI: 10/15 Healthy controls: 13/22 *Validation* Male/female AD: 2/5 aMCI: 5/5 Healthy controls: 5/5	Methylguanosine, histidylphenylalanine, choline-cytidine AD/healthy controls: AUC (discovery and validation) = 1.00 Sensitivity: 100% Specificity: 100% *p* value < 0.01 Amino-dihydroxybenzene, glucosyl-galactosyl-hydroxylysine-H_2_O, aminobytyric acid + H_2_ AD/aMCI: AUC (discovery and validation) = 1.00 Sensitivity: 100% Specificity: 100% *p* value < 0.01 Phenylalanylproline, phenylalanylphenylalanine, urocanic acid AD/healthy controls: AUC discovery: 0.820 AUC validation: 0.814 Sensitivity: 71.4% Specificity: 90.0% *p* value < 0.01 Alanylphenylalanine, phenylalanylproline AD/aMCI: AUC discovery: 0.881 AUC validation: 0.786 Sensitivity: 71.4% Specificity: 80.0% *p* value < 0.01

Ashton et al. [15]	t-tau	SIMOA	AD: *n* = 53 aMCI: *n* = 68 Healthy controls: *n* = 160	Mean age AD: 81.4 ± 6.6 aMCI: 79.8 ± 7.4 Healthy controls: 78.0 ± 6.7	Male/female AD: 23/30 aMCI: 33/35 Healthy controls: 66/94	↑ t-tau, although not statistically significant, *p* value AD/aMCI/healthy controls = 0.219 Correlation with ventricular volume for AD: *p* value = 0.045

Pekeles et al. [26]	t-tau p-tau	Western blot	Two studies (round one and round two) *Round one* AD: *n* = 46 aMCI: *n*=55 Healthy controls: *n* = 47 *Round two* AD: *n* = 41 FTD: *n* = 16 Healthy older controls: *n* = 44 Neurological patients (other than dementia): *n* = 12 Young healthy controls: *n* = 76	Two studies (round one and round two) *Round one* Median age (IQR) AD: 80 (9) aMCI: 78 (14) Healthy controls: 73 (6) *Round two* Median age (IQR) AD: 80 (8) FTD: 71.5 (10) Healthy older controls: 72 (7) Neurological patients (other than dementia): 55 (11) Young healthy controls: 32 (22)	Two studies (round one and round two) *Round one* Male/female AD: 24/22 aMCI: 23/32 Healthy controls: 15/32 *Round two* Male/female AD: 17/24 FTD: 11/5 Healthy older controls: 14/30 Neurological patients (other than dementia): 5/7 Young healthy controls: 31/45	Two studies (round one and round two) *Round one* ↑ p-tau/t-tau at phosphorylation site: S396, S404, and the combination of S400, T403, and T404, *p* value < 0.05 *Round two* ↑ median p-tau/t-tau at phosphorylation site: S396, *p* value AD/healthy older controls < 0.05 Sensitivity S396: 73% Specificity S396: 50% Sensitivity S404: 83% Specificity S404: 30%

McGeeR et al. [13]	A*β*42	ELISA	AD: *n* = 23 Low controls: *n* = 25 High controls (risk for AD): *n* = 6	Mean age AD: 71.3 Low controls: 54.2 High controls (risk for AD): 69	Male/female AD: 8/5 Low controls: 17/8 High controls (risk of AD): 3/3	↑ A*β*42 (AD > high controls (risk for AD) > low controls) *p* value AD/low controls/high controls < 0.001

Bakhtiari et al. [16]	AchE activity	Ellman's colorimetric method	AD: *n* = 15 Healthy controls: n=15	Mean age AD: 78.4 Healthy controls: 71	Male/female AD: 9/6 Healthy controls: 7/8	↑ AchE activity, *p* value AD/control = 0.25

Carro et al. [27]	Lactoferrin	ELISA	Two studies (discovery and validation) *Discovery* AD: *n* = 80 aMCI: *n* = 44 Healthy controls: *n* = 91 PD: *n* = 59 *Validation* AD: *n* = 36 aMCI: *n* = 15 Healthy controls: *n* = 40	Two studies (discovery and validation) *Discovery* Mean age AD: 76.2 ± 5.33 aMCI: 75.16 ± 5.13 Healthy controls: 73.7 ± 6.88 PD: 69.5 ± 8.6 *Validation* Mean age AD: 80.67 ± 8.67 aMCI: 68.93 ± 6.12 Healthy controls: 66.78 ± 7.33	Two studies (discovery and validation) *Discovery* Male/female AD: 31/49 aMCI: 19/25 Healthy controls: 32/59 PD: 27/32 *Validation* Male/female AD: 13/23 aMCI: 10/5 Healthy controls: 15/25	Two studies (discovery and validation) *Discovery* ↓ lactoferrin, *p* value AD/healthy controls < 0.001, *p* value aMCI/healthy controls < 0.001 *Validation* Sensitivity: 100%, specificity: 100% Correlation with CSF A*β*42: *p* value < 0.001 Correlation with CSF t-tau: *p* value < 0.001 Correlation with MMSE in AD and aMCI: *p* value < 0.001

Yilmaz et al. [17]	Metabolites (propionate, acetone)	Proton NMR spectroscopy	AD: *n* = 9 aMCI: *n* = 8 Healthy controls: *n* = 12	Mean age AD: 85 ± 7 aMCI: 83 ± 5 Healthy controls: 82 ± 8	Male/female AD: 3/6 aMCI: 3/5 Healthy controls: 4/8	↑ propionate, *p* value AD/aMCI/healthy controls = 0.034 Regression model for propionate and acetone AD/healthy controls: AUC = 0.871 Sensitivity: 90.9 % Specificity: 84.2%

Lee et al. [12]	A*β*42	ELISA	AD+pre-AD: *n* = 10 Healthy controls: *n* = 26 PD: *n* = 1	Mean age AD+pre-AD: 70.1 Healthy controls+PD (control): 54.6	Male/female AD+pre-AD: 3/7 Healthy controls+PD (control): 18/9	↑ A*β*42, *p* value AD/control < 0.001

Liang et al. [18]	Spinganine-1-phosphate Ornithine Phenyllactic acid Inosine 3-dehydrocarnitine Hypoxanthine	FUPLC-MS	AD: *n*=256 Healthy controls: *n* = 218	Mean age AD: 78.6 ± 6.8 Healthy controls: 77.9 ± 5.6	Male/female AD: 124/132 Healthy controls: 102/116	↑ spinganine-1-phosphate, *p* value < 0.01 Sensitivity: 99.4% Specificity: 98.2% ↑ ornithine, *p* value < 0.01 Sensitivity: 81.9% Specificity: 90.7% ↑ phenyllactic acid, *p* value < 0.01 ↓ inosine, *p* value < 0.01 ↓ 3-dehydrocarnitine, *p* value < 0.01 ↓ hypoxanthine, *p* value < 0.01

Lau et al. [19]	Trehalose A*β*42 t-tau p-tau	Trehalose: EG-IDFET biosensor A*β*42, t-tau, p-tau: ELISA	AD: *n* = 20 Healthy controls: *n* = 20 PD: *n* = 20	Mean age AD: 72.5 ± 7.68 Healthy controls: 66.1 ± 7.79 PD: 73 ± 8.07	Male/female AD: 8/12 Healthy controls: 9/11 PD: 5/15	Trehalose concentration: AD > PD > healthy controls A*β*42: not detected t-tau: no significant differences ↑ p-tau

Kim et al. [21]	A*β*42 A*β*40	Immunoassay with nanobeads	AD: *n* = 28 Healthy controls: *n* = 17	N.A.	N.A.	↑ A*β*42 (statistically significant) ↑ A*β*40 (not statistically significant)

Shi et al. [24]	A*β*42 t-tau p-tau	ELISA (Luminex assay)	AD: *n* = 21 Healthy controls: *n* = 38	Mean age AD: 68.8 Healthy controls: 69	Male/female AD: 10/11 Healthy controls: 19/19	A*β*42: not detected ↑ t-tau ↑ p-tau ↑ p-tau/t-tau in AD patients, *p* value < 0.05

Bermejo-Pareja et al. [20]	A*β*42 A*β*40	ELISA	AD: *n* = 70 (mild: *n* = 29, moderate: *n* = 24, severe: *n* = 17) Healthy controls: *n* = 56 PD: *n* = 51	Mean age AD: 77.2 (60-91) Healthy controls: 74.35 (64-85) PD: 72.96 (60-93)	Male/female AD: 21/49 Healthy controls: 17/39 PD: 26/25	A*β*42: ↑ concentration in mild and moderate AD *p* value mild AD = 0.043 *p* value AD/healthy control < 0.05 Sensitivity: 16% Specificity: 93% Interaction with age: *p* value = 0.016 Interaction with gender: *p* value = 0.002 A*β*40: ↑ concentration in AD and PD

Boston et al. [22]	AchE	Ellman's colorimetric method	AD: *n* = 15 Healthy controls: *n* = 13 VaD: *n* = 13	Mean age AD: 83.5 (71.3-95.8) Healthy controls: 80.8 (70.8-92.1) VaD: 81.8 (71.3-93.3)	Male/female AD: 5/10 Healthy controls: 7/6 VaD: 9/4	No significant difference

Sayer et al. [23]	AchE activity	Ellman's colorimetric method	AD responders: *n* = 22 AD nonresponders: 14 Healthy controls: *n* = 11	Mean age AD responders: 75 (60-86) AD nonresponders: 75 (65-87) Healthy controls: 71 (64-91)	Male/female AD responders: 7/15 AD nonresponders: 4/10 Healthy controls: 6/5	↓ AchE activity, *p* value nonresponders/healthy controls < 0.005 Interaction with age in controls: *p* value < 0.001

AD: Alzheimer's disease. aMCI: amnestic mild cognitive impairment. PD: Parkinson disease. FTD: frontotemporal dementia. A*β*42: amyloid beta 1-42. A*β*40: amyloid beta 1-40. p-tau: phosphorylated tau. t-tau: total tau. AchE: acetylcholinesterase. CSF: cerebrospinal fluid. MMSE: minimental state examination. ELISA: enzyme-linked immunosorbent assay. SIMOA: single molecule array. LC-MS: liquid chromatography mass spectrometry. FUPLC-MS: fast ultraperformance liquid chromatography mass spectrometry. EG-IDFET biosensor: extended gate ion-sensitive field-effect transistor biosensor. ↑: increased levels of the biomarker in patients with AD. ↓: decreased levels of the biomarker in patients with AD. AUC: area under the curve. N.A.: not available.
